# Newborn Screening with (C16 + C18:1)/C2 and C14/C3 for Carnitine Palmitoyltransferase II Deficiency throughout Japan Has Revealed C12/C0 as an Index of Higher Sensitivity and Specificity

**DOI:** 10.3390/ijns9040062

**Published:** 2023-10-27

**Authors:** Go Tajima, Keiichi Hara, Miyuki Tsumura, Reiko Kagawa, Fumiaki Sakura, Hideo Sasai, Miori Yuasa, Yosuke Shigematsu, Satoshi Okada

**Affiliations:** 1 Division of Neonatal Screening, Research Institute, National Center for Child Health and Development, 2-10-1 Okura, Setagaya-ku, Tokyo 157-8535, Japan; 2 Department of Pediatrics, Hiroshima University Graduate School of Biomedical and Health Sciences, 1-2-3 Kasumi, Minami-ku, Hiroshima 734-8551, Japan; hara-keiichi@kxd.biglobe.ne.jp (K.H.); m055@hiroshima-u.ac.jp (M.T.); rekagawa@hiroshima-u.ac.jp (R.K.); fsakura@kazusa.or.jp (F.S.); sokada@hiroshima-u.ac.jp (S.O.); 3 Department of Pediatrics, National Hospital Organization Kure Medical Center and Chugoku Cancer Center, 3-1 Aoyama-cho, Kure 737-0023, Japan; 4 Department of Technology Development, Kazusa DNA Research Institute, Kisarazu 292-0818, Japan; 5 Department of Early Diagnosis and Preventive Medicine for Rare Intractable Pediatric Diseases, Graduate School of Medicine, Gifu University, 1-1 Yanagido, Gifu 501-1194, Japan; sasai.hideo.f1@f.gifu-u.ac.jp; 6 Department of Pediatrics, Faculty of Medical Sciences, University of Fukui, 23-3 Matsuoka-Shimoaizuki, Eiheiji-cho, Fukui 910-1193, Japan; miori@u-fukui.ac.jp (M.Y.); yosuke3321181@gmail.com (Y.S.)

**Keywords:** carnitine palmitoyltransferase II deficiency, CPT2, newborn screening, false-positive, tetradecanoylcarnitine (C14), dodecanoylcarnitine (C12), acylcarnitine ratios

## Abstract

Carnitine palmitoyltransferase (CPT) II deficiency is a long-chain fatty acid oxidation disorder. It manifests as (1) a lethal neonatal form, (2) a hypoglycemic form, or (3) a myopathic form. The second form can cause sudden infant death and is more common among Japanese people than in other ethnic groups. Our study group had earlier used (C16 + C18:1)/C2 to conduct a pilot newborn screening (NBS) study, and found that the use of C14/C3 for screening yielded lower rates of false positivity; in 2018, as a result, nationwide NBS for CPT II deficiency started. In this study, we evaluated the utility of these ratios in 71 NBS-positive infants and found that the levels of both C14/C3 and (C16 + C18:1)/C2 in patients overlapped greatly with those of infants without the disease. Among the levels of acylcarnitines with various chain lengths (C18 to C2) and levels of free carnitine (C0) as well as their ratios of various patterns, C12/C0 appeared to be a promising index that could reduce false-positive results without missing true-positive cases detected by current indices. Although some cases of the myopathic form may go undetected even with C12/C0, its use will help prevent life-threatening onset of the hypoglycemic form of CPT II deficiency.

## 1. Introduction

Carnitine palmitoyltransferase (CPT) II is an enzyme bound to the mitochondrial inner membrane. Long-chain fatty acids are transported into the mitochondria as acylcarnitines with corresponding chain lengths via the sequential function of acyl–coenzyme A (CoA) synthetase, CPT I, and carnitine-acylcarnitine translocase (CACT). These long-chain acylcarnitines, represented by palmitoylcarnitine (C16), are then turned back into acyl-CoA by CPT II to supply substrates for the β-oxidation system. CPT II deficiency has been clinically classified into three phenotypes: (1) a lethal neonatal form associated with cardiomyopathy; (2) a hypoglycemic form, manifesting mainly during infancy and early childhood, that provokes hypoglycemia, Reye-like encephalopathy, and, in the most severe cases, cardiopulmonary arrest; and (3) a myopathic form characterized by recurrent rhabdomyolysis with onset in adolescence or later. Among populations of European descent, the myopathic form accounts for most cases of CPT II deficiency because one variant, c.338C > T (p.S113L), is highly prevalent [1]. In contrast, the hypoglycemic form appears more commonly among Japanese populations. According to previous reports, c.1148T > A (p.F383Y) was frequently detected in Japanese patients exhibiting acute hypoglycemic crisis, including cases of sudden infant death [2]. As a result, an effective program of newborn screening (NBS) for CPT II deficiency was strongly expected in Japan.

Tandem mass spectrometry (MS/MS)–based NBS was introduced in Japan in 1997 in a pilot study in which CPT II deficiency was screened with, since 2004, C16 and C18:1 as indices. In 2010, a 7-month-old boy presented with acute encephalopathy associated with hypoketotic hypoglycemia, hyperammonemia, and marked elevation of serum creatine kinase, which resulted in severe neurological sequelae. The diagnosis of CPT II deficiency was confirmed [3]. In the pilot study, the patient had passed NBS with MS/MS. On the basis of his NBS data, in 2011, we adopted the ratio of C16 + C18:1 to acetylcarnitine (C2) as an alternative index and continued the pilot study. In the subsequent 6 years, positive results of this ratio were detected in 4 patients with CPT II deficiency, 1 carrier heterozygous for a pathogenic variant of the causative *CPT2* gene, and 13 normal infants [4].

A case report published in 2017 concerned a 4-year-old girl presenting with rhabdomyolysis, in whom CPT II deficiency had been missed in NBS, which suggested that the ratio of several long-chain acylcarnitines to propionylcarnitine (C3) in dried blood specimens (DBSs) from newborns, such as (C16 + C18:1)/C3, C18/C3, C16/C3, C16-OH/C3, and C14/C3, can serve as better indices in CPT II deficiency screening [5]. Analysis of the pilot study data of 21 NBS-positive infants revealed that C14/C3 was a promising index with equal sensitivity and lower rates of false positivity in comparison with (C16 + C18:1)/C2 [4]. Our study group therefore proposed the use of (C16 + C18:1)/C2 and C14/C3 in NBS for CPT II deficiency, and this disease was added to the official panel of nationwide NBS in 2018 [6]. Since then, however, the number of false-positive results has gradually increased, even with the use of C14/C3.

In this study, we evaluated the utility of these indices in comparison with various acylcarnitines and their ratios on a larger scale, with the aim of discovering more effective NBS indices for CPT II deficiency.

## 2. Materials and Methods

### 2.1. Screening Test for CPT II Deficiency

DBSs for NBS were generally collected on postnatal day 4 or 5, in accordance with the official protocol used since the start of NBS for amino acid disorders in 1977, in which a uniform filter paper (Advantec Toyo Kaisha, Tokyo, Japan) has been used. Indices for CPT II deficiency were as follows: (1) During the pilot study from 2004 to 2010, C16 ≥ 6.3 nmol/mL and C18:1 ≥ 3.6 nmol/mL were used; both cutoff values corresponded to the means + 4 standard deviations (SD) of values in healthy control newborns when they were set [4]; (2) during the pilot study from 2011 to 2017, the (C16 + C18:1)/C2 cutoff value was ≥0.62 (99.9th percentile in healthy controls) and the C16 cutoff value was ≥3.0 nmol/mL (79.5th percentile in healthy controls) [4]; and (3) after the start of nationwide NBS in April 2018, cutoffs for both (C16 + C18:1)/C2 and C14/C3 were set at the values for the 99.9th percentile in healthy controls [6]. The nationwide NBS tests have been assigned to 35 regional laboratories, where the mean cutoffs used were 0.432 ± 0.073 for (C16 + C18:1)/C2 and 0.386 ± 0.077 for C14/C3. With regard to tandem mass spectrometers and sample preparation kits, including stable-isotope-labeled internal standards, products of several manufacturers have been used in various combinations. Inter-laboratory variations are managed within acceptable ranges by the Quality Control Committee of the Japanese Society for Neonatal Screening.

### 2.2. Confirmatory Tests for CPT II Deficiency

At the first examination of the NBS-positive infants, acylcarnitine profiles in their serum were analyzed. To confirm the findings, we measured the enzymatic activity of CPT II as described in a previous report [4]. Using high-performance liquid chromatography, we detected the production of palmitoyl-CoA from palmitoyl-l-carnitine and CoA trilithium salt catalyzed by a crude lysate of peripheral lymphocytes. Lymphocytes were sonicated in 1% octyl glucoside solution to abolish the activity of CPT I.

For several NBS-positive infants who were screened before measurement of CPT II activity was established, we measured fatty acid oxidation, also as described in previous reports [4,7]. In brief, peripheral mononuclear cells were incubated with l-carnitine and deuterium-labeled palmitate (d_31_-palmitate) and then washed and homogenized. The supernatant was analyzed by flow-injection electrospray ionization MS/MS, fatty acid oxidation ability was assessed according to the ratio of d_1_-acetylcarnitine (d_1_C2) to d_31_-palmitoylcarnitine (d_31_C16), and CPT II activity was assessed according to the ratio of d_27_-tetradecanoylcarnitine (d_27_C14) to d_31_C16.

In cases in which CPT II activity was judged to be impaired, the results were subjected to genetic analysis if informed consent was obtained from the infants’ parents. Genomic DNA was extracted from peripheral white blood cells. All exons and flanking intron regions containing *CPT2* were amplified with polymerase chain reaction, and the products were sequenced directly. Because the biochemical characteristics of CPT II deficiency are similar to those of CACT deficiency, sequencing of the gene responsible for the latter (*SLC25A20*) was also performed for some infants who were normal with CPT II and for whom further exclusion diagnosis was judged to be necessary. In several cases, gene panel sequencing provided by the national health insurance scheme that targeted fatty acid oxidation disorders (FAODs) was performed according to the choice of doctors in charge.

### 2.3. Receiver Operating Characteristic Analysis

Receiver operating characteristic (ROC) curves were generated to determine the sensitivity and specificity of various acylcarnitines and their ratios; for this task, we used statistical software (R version 4.2.2 and R studio; The R Foundation, Vienna, Austria). To determine the optimal cutoff for each item, we used the Youden index.

## 3. Results

### 3.1. Diagnoses of NBS Positivity and Levels of the Current Indices

The characteristics of NBS-positive infants enrolled in this study are summarized in Table 1. The results of NBS and confirmatory tests for each infant are listed in Supplementary Table S1 in the order of CPT II activity, and they were classified as follows.

Group A included 20 infants in whom CPT II deficiency was confirmed (N-01 to N-20). In addition, the aforementioned case of the 7-month-old boy with hypoglycemic encephalopathy that was missed in the pilot NBS (S-01) was also assigned to this group because the current index (C16 + C18:1)/C2 was adopted based on his NBS data.

Group B included 17 infants. Two (group B-1: N-21 and N-37) were confirmed to be carriers heterozygous for pathogenic variants of the causative *CPT2* gene, and three (group B-2: N-22, N-26, and N-35) were homozygous for a thermolabile polymorphism, c.1055T > G (p.F352C); CPT II activity in these five infants ranged from 31.8% to 70.8%. In 12 infants (group B-3: N-23 to N-25, N-27 to N-34, and N-36), CPT II activity was also within that range; these 12 were suspected to be heterozygous for pathogenic variants of the causative *CPT2* gene, although genetic analysis conducted in 5 of them did not reveal this genotype. The other seven did not undergo genetic testing because their parents did not consent to further confirmatory tests.

Group C included 33 infants (N-38 to N-70) whose CPT II activity was 78.1% or higher; they were judged to be normal. Results of genetic analysis conducted in 12 of them were consistent with their CPT II activity.

**Figure 1:**
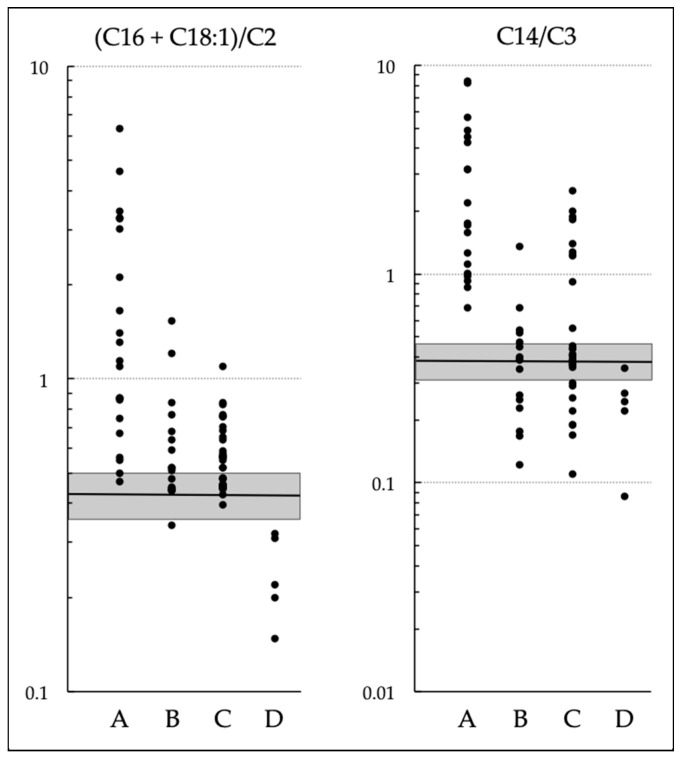
Levels of indices in dried blood specimens from newborns. A–C: Newborn screening (NBS)–positive infants: patients with carnitine palmitoyltransferase (CPT) II deficiency (A); carriers heterozygous for pathogenic variants, infants homozygous for p.F352C, and other infants with carrier-level CPT II activity (B); and infants with normal-level CPT II activity (C). D: Patients with symptoms of CPT II deficiency but normal NBS results. Shaded squares indicate ranges of cutoffs (means ± SD) set at the 99.9th percentiles in 35 regional laboratories: 0.432 ± 0.073 for (C16 + C18:1)/C2 and 0.386 ± 0.077 for C14/C3. Several values of C14/C3 below cutoff values in groups B and C correspond to levels in newborns who were screened before this index was adopted.

Levels of (C16 + C18:1)/C2 and C14/C3 in groups A, B, and C are illustrated in Figure 1. Because the study included data from newborns who were screened before C14/C3 was adopted, several values of C14/C3 in groups B and C are below the cutoff. An additional group, D, is described in the next section.

### 3.2. Patients with Symptoms of CPT II Deficiency but Normal NBS Results

We confirmed diagnoses of CPT II deficiency in several patients with symptoms of CPT II deficiency who had passed NBS. Data required for this were available from five patients who presented with rhabdomyolysis without hypoglycemia (S-02 to S-06; Table 1 and Table S1). The results of tests for (C16 + C18:1)/C2 or C14/C3, or both, in their DBSs were judged to be normal according to cutoff values in each regional laboratory. These patients were designated as group D, and levels of their NBS indices that were checked after the diagnoses are illustrated in Figure 1.

### 3.3. Comparison of (C16 + C18:1)/C2 and C14/C3 with Various Acylcarnitines and Their Ratios

**Figure 2:**
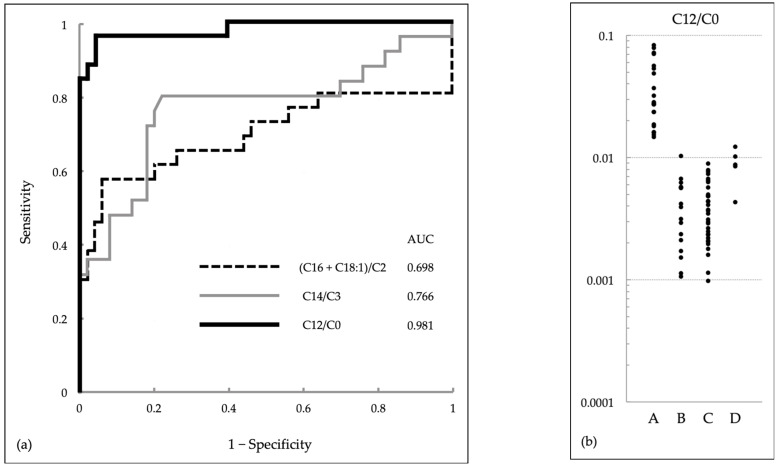
(**a**) Receiver operating characteristic (ROC) curve of C12/C0 in comparison with those of the current newborn screening (NBS) indices (C16 + C18:1)/C2 and C14/C3. The area under the curve (AUC) for C12/C0 was 0.981 (95% confidence interval, 0.9497~1). (**b**) Levels of C12/C0 in dried blood specimens from newborns. A, B, and C refer to NBS-positive infants: patients with carnitine palmitoyltransferase (CPT) II deficiency (A); carriers heterozygous for pathogenic variants, infants homozygous for p.F352C, and other infants with carrier-level CPT II activity (B); and infants with normal-level CPT II activity (C). D refers to patients with symptoms of CPT II deficiency but normal NBS results.

Concentrations of individual acylcarnitines (C16, C18:1, C18, C16-OH, C14, C14:1, C12, C10, C8, C6, C4, C3, C2) and free carnitine (C0) in DBSs from newborns in our study are listed in Supplementary Table S2 and Supplementary Figure S1. Although none of these single items can clearly differentiate patients with CPT II deficiency (group A) from infants without the disease (groups B and C), overlap between these groups appears to be comparatively less for C12 than for the other acylcarnitines. According to ROC analysis, sensitivity, specificity, and area under the curve (AUC) were highest for C12, followed by C14 and C10 (Table 2). Among acylcarnitines of chain lengths shorter than C10 and free carnitine, C2 and C0 were similarly expected as denominators that could improve the sensitivity and specificity of C12. ROC analysis of ratios of C14, C12, or C10 to C2 or C0, in comparison with (C16 + C18:1)/C2 and C14/C3, revealed that C12/C0 is the only ratio that is superior to C12 concentration in sensitivity (0.962 vs. 0.885), specificity (0.958 vs. 0.938), and AUC (0.981 vs. 0.960; Table 3). The superiority of C12/C0 to (C16 + C18:1)/C2 and C14/C3 is evident from their ROC curves (Figure 2a). The use of C12/C0 can distinguish group A from groups B and C and may have helped identify four of the five patients in group D as NBS-positive infants (Figure 2b).

### 3.4. Results of Serum Acylcarnitine Analysis at the First Medical Examination

**Figure 3:**
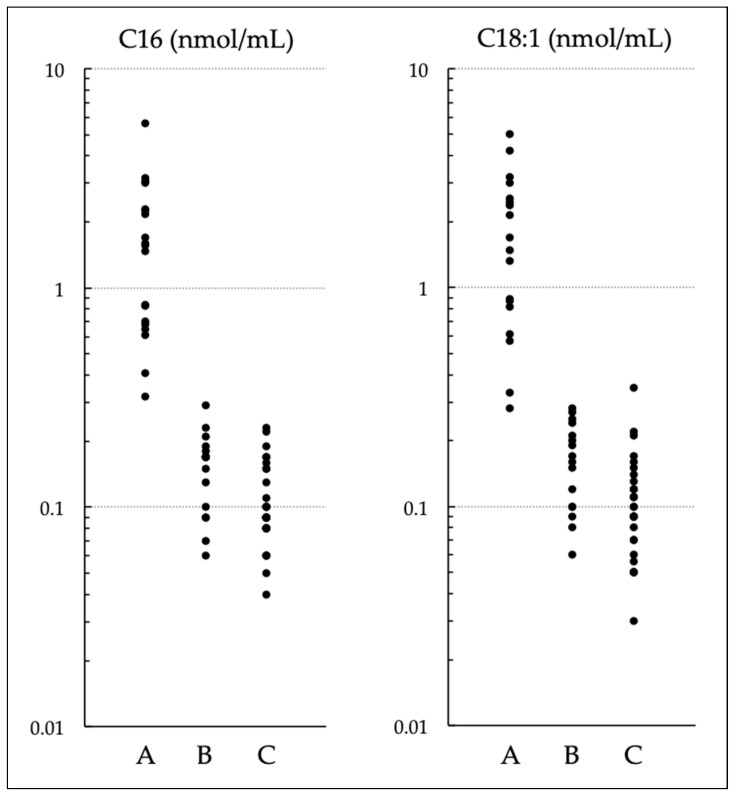
Levels of C16 and C18:1 in serum of newborn screening (NBS)–positive infants: patients with carnitine palmitoyltransferase (CPT) II deficiency (A); carriers heterozygous for pathogenic variants, infants homozygous for p.F352C, and other infants with carrier-level CPT II activity (B); and infants with normal-level CPT II activity (C).

Levels of C16 and C18:1 in serum of NBS-positive infants are illustrated in Figure 3. The samples were generally obtained at the first medical examinations, which were within 1 month after birth in most cases. As shown in a previous report [4], these indices are apparently quite useful in differentiating between group A and groups B and C.

## 4. Discussion

Although CPT II deficiency and very-long-chain acyl-CoA dehydrogenase (VLCAD) deficiency have similar clinical phenotypes, research articles focusing on NBS for CPT II deficiency are apparently far fewer than those for VLCAD. One reason for this discrepancy is that in countries where NBS for CPT II deficiency has been conducted, the prevalence of CPT II deficiency is low [8]; however, this may be explained partially by the difficulty in detecting the myopathic form caused by the mild variant p.S113L that is prevalent among populations of European descent [1]. According to another previous report, the disease frequency shown by NBS conducted in Australia, Germany, and the United States ranged from 1/380,000 to 1/2,000,000 newborns [9].

Even after the ratios of long-chain acylcarnitines to C3, especially C14/C3, were proposed as promising indices for NBS in 2017 [4,5], we found only two articles in which data for C14/C3 are presented: one single-case report from Canada [10] and one about a study on NBS for carnitine cycle disorders conducted in a province of China [11]. In the latter article, decreases in free carnitine (C0) or C0/(C16 + C18) levels or elevated levels of C12 to C18:1 were used as indices for detecting CPT II deficiency, and four cases were identified. As additional indices, (C16 + C18:1)/C2, (C16 + C18:1)/C3, C16/C3, C16:1/C3, C18/C3, C18:1/C3, and C14/C3 were clearly elevated in all four patients. However, no information on false-positive results was included in this report.

In contrast, the burden of CPT II deficiency is apparently heavier in Japan. A former nationwide survey on symptomatic FAODs diagnosed or reported between 1985 and 2000 revealed that the largest number of affected patients had CPT II deficiency [12]. In the pilot study of MS/MS-NBS conducted from 2004 to 2012 in several areas of Japan, CPT II deficiency was diagnosed in 7 (including S-01, N-02, N-03, N-05, N-06, and N-07 in our study) of 1,740,387 newborns, which represented a frequency of 1/248,627. With regard to clinical phenotypes, the survey conducted before the start of nationwide NBS for CPT II deficiency revealed 13 cases in which patients younger than 2 years died suddenly and the diagnoses were clarified afterward [unpublished data]. Data on genotypes of the NBS-positive patients in our study also indicate the predominance of the hypoglycemic form of the disease: c.1148T > A (p.F383Y) and c.520G > A (p.E174K), both known to cause sudden infant death [2,13], together with two other frameshift variants, account for 26 of 42 variant alleles.

In a report published in 2017, NBS data revealed that 22 infants (including S-01, N-02, N-03, N-05, N-06, N-07, N-10, N-12, N-21, N-26, N-36, N-37, N-43, N-47, N-50, N-52, N-54, N-55, N-56, N-61, N-63, and N-66 in our study) had positive results for (C16 + C18:1)/C2, and C14/C3 helped distinguish 8 patients with CPT II deficiency from 14 infants without the disease [4]. However, since 2018, when the nationwide NBS for CPT II deficiency started, the number of false-positive results for not only (C16 + C18:1)/C2 but also C14/C3 increased, many of which were caused by very low levels of C3 (Supplementary Figure S1). Results of our study, according to the data of a larger scale, indicated that C12/C0 is apparently the most effective NBS index for CPT II deficiency that can reduce false-positive results without missing true-positive cases detected by the current indices.

We diagnosed CPT II deficiency in several patients with symptoms of the disease who had passed the nationwide NBS. Five of them were enrolled in this study, and NBS with C12/C0 and the optimal cutoff might have identified four of them. However, analysis of the data of those patients suggested that in some newborns with CPT II deficiency, the disease is difficult to detect by MS/MS-NBS, even with C12/C0. Because the accumulation of acylcarnitines indicative of FAODs tends to be smaller in well-fed states, sampling of DBS on postnatal day 4 or 5 may be late for some patients with milder phenotypes, which appears to be especially true of CPT II deficiency in comparison with VLCAD deficiency, for which NBS can identify many newborns with the mildest disease [14]. Fortunately, none of the patients with false-negative results and myopathic symptoms of CPT II deficiency whom we have studied so far have had hypoglycemia. In general, myopathic forms of long-chain FAODs are not fatal and do not result in severe sequelae, but complete control of such symptoms is difficult [15]. Therefore, NBS may not be appropriate for detecting the myopathic form of CPT II deficiency. As far as we know, no patient with the hypoglycemic form has passed the nationwide NBS for CPT II deficiency in Japan. By comparison, sampling of DBS is scheduled from 24 h to 48 h after birth in many countries [15], where the results of this study need further validation.

## 5. Conclusions

Our study group has conducted NBS for CPT II deficiency as a pilot study since 2004 and as part of the nationwide healthcare service since 2018, in Japan, where the potentially fatal hypoglycemic form is apparently predominant, and the disease has been confirmed in 20 of 70 NBS-positive infants. Approximately 60% of the *CPT2* variant alleles of these patients with CPT II deficiency were actually indicative of the hypoglycemic form. Because of the increasing number of false-positive results, we compared levels of acylcarnitines with various chain lengths (C18 to C2) and levels of free carnitine (C0) thoroughly, as well as the ratios of various patterns in the DBSs of newborns. As a result, C12/C0 has appeared to be the most effective diagnostic index. We plan to conduct nationwide studies to evaluate its performance both retrospectively and prospectively, with the aim of setting an appropriate cutoff. Substitution of the current indices (C16 + C18:1)/C2 and C14/C3 with C12/C0 is expected to reduce the number of NBS results that are falsely positive for CPT II deficiency in Japan, while adequate sensitivity for the hypoglycemic form will be retained.

## Figures and Tables

**Table 1:** Classification of infants enrolled in this study.

Groups and Descriptions	*n*	Case ID *	CPT II Activity
Group A: NBS-positive, patients with CPT II deficiency	21	S-01, N-01 to N-20	1.2–27.9%
Group B-1: NBS-positive, carriers heterozygous for pathogenic *CPT2* variants	2	N-21, N-37	31.8%, 70.8%
Group B-2: NBS-positive, homozygous for p.F352C	3	N-22, N-26, N-35	34.0%, 52.0%, 63.8%
Group B-3: NBS-positive, CPT II activity between 31.8% and 70.8% without genetic confirmation of either heterozygosity for pathogenic variants or homozygosity for p.F352C	12	N-23 to N-25, N-27 to N-34, N-36	37.7–67.4%
Group C: NBS-positive infants with normal-level CPT II activity	33	N-38 to N-70	78.1–410.4% (mean value = 152.2% ± 83.8%)
Group D: Patients with symptoms of CPT II deficiency but normal results in NBS	5	S-02 to S-06	8.1–30.2%

Abbreviations: CPT II, carnitine palmitoyltransferase II; NBS, newborn screening. * Detailed data of individual infants are presented in Supplementary Tables S1 and S2.

**Table 2:** Results of receiver operating characteristic analysis of individual acylcarnitines and free carnitine.

Characteristic	Acylcarnitine and Free Carnitine (C0) Levels													
	C16	C18:1	C18	C16-OH	C14	C14:1	C12	C10	C8	C6	C4	C3	C2	C0
Optimal cutoff (nmol/mL)	9.8	2.56	1.695	0.027	0.295	0.106	0.155	0.085	0.095	0.02	0.111	0.55	5.61	20.225
Sensitivity	0.231	0.346	0.308	0.600	0.640	0.400	0.885	0.846	0.192	0.696	0.750	0.808	0.769	0.885
Specificity	1	0.980	0.980	0.872	0.900	0.851	0.938	0.646	0.917	0.425	0.475	0.480	0.640	0.620
Area under the curve	0.526	0.576	0.603	0.727	0.822	0.644	0.960	0.784	0.524	0.526	0.600	0.622	0.706	0.733

**Table 3:** Results of receiver operating characteristic analysis of acylcarnitine ratios.

Characteristic	Acylcarnitine Ratios							
	(C16 + C18:1)/C2	C14/C3	C14/C2	C14/C0	C12/C2	C12/C0	C10/C2	C10/C0
Optimal cutoff	0.857	0.620	0.066	0.018	0.020	0.008	0.017	0.005
Sensitivity	0.577	0.800	0.760	0.800	0.885	0.962	0.808	0.962
Specificity	0.940	0.780	1	0.940	0.979	0.958	0.896	0.667
Area under the curve	0.698	0.766	0.832	0.908	0.932	0.981	0.895	0.885

## Data Availability

The data presented in this study are available on request from the corresponding author.

## Supplementary Materials

The following supporting information can be downloaded at: https://www.mdpi.com/article/10.3390/ijns9040062/s1, Table S1: Results of newborn screening (NBS) and confirmatory tests of the infants enrolled in this study; Table S2: Concentrations of acylcarnitines and free carnitine (C0) in dried blood specimens from newborns; Figure S1: Concentrations of acylcarnitines and free carnitine (C0) in dried blood specimens from newborns.
