# Supplementary material for: Newborn Screening with (C16 + C18:1)/C2 and C14/C3 for Carnitine Palmitoyltransferase II Deficiency throughout Japan Has Revealed C12/C0 as an Index of Higher Sensitivity and Specificity
Source: Int J Neonatal Screen. 2023 Oct 27;9(4):62. doi: 10.3390/ijns9040062 (PMC10660675; doi:10.3390/ijns9040062)
Supplement: Supplementary file 1 [file IJNS-09-00062-s001.zip › IJNS2023_FigureS1.pdf]

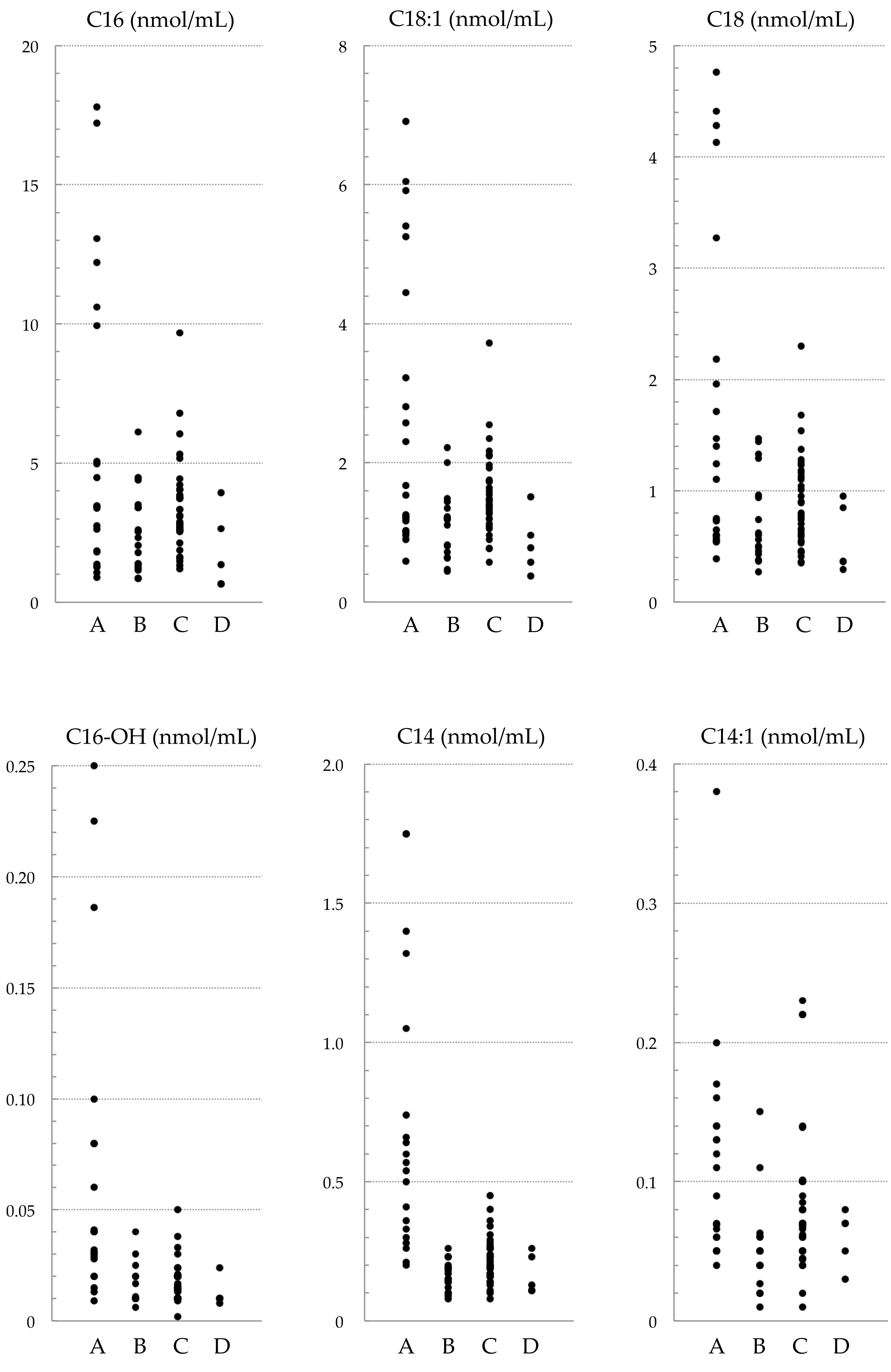

C16 (nmol/mL)
0
5
10
15
20
C18:1 (nmol/mL)
0
2
4
6
8
C18 (nmol/mL)
0
1
2
3
4
5
C16-OH (nmol/mL)
0
0.05
0.10
0.15
0.20
0.25
C14 (nmol/mL)
0
0.5
1.0
1.5
2.0
C14:1 (nmol/mL)
0
0.1
0.2
0.3
0.4
A
B
C
D

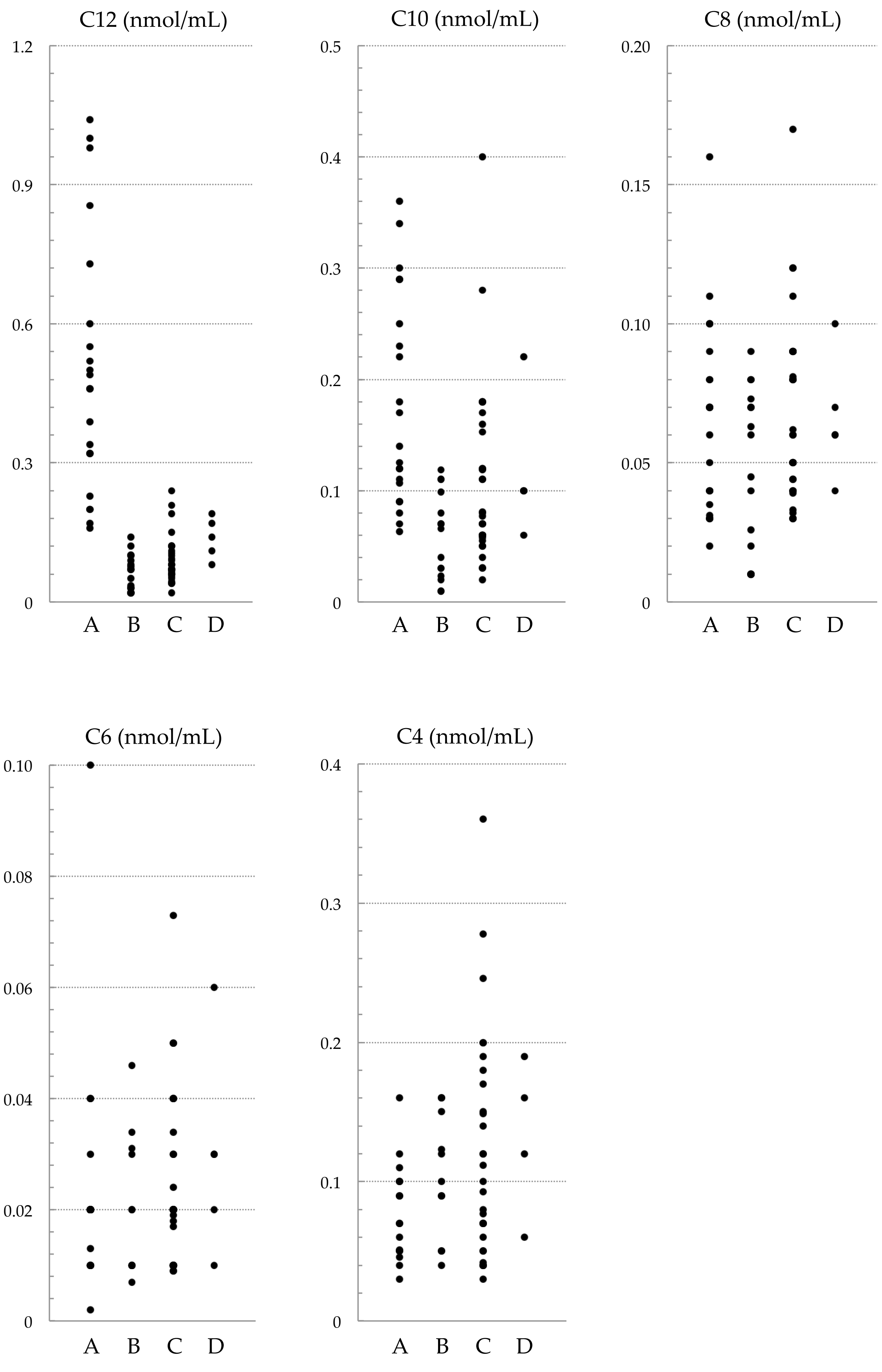

C12 (nmol/mL)
C10 (nmol/mL)
C8 (nmol/mL)
C6 (nmol/mL)
C4 (nmol/mL)
0
0.3
0.6
0.9
1.2
0.1
0.2
0.3
0.4
0.5
0.05
0.10
0.15
0.20
0.02
0.04
0.06
0.08
0.10
A
B
C
D

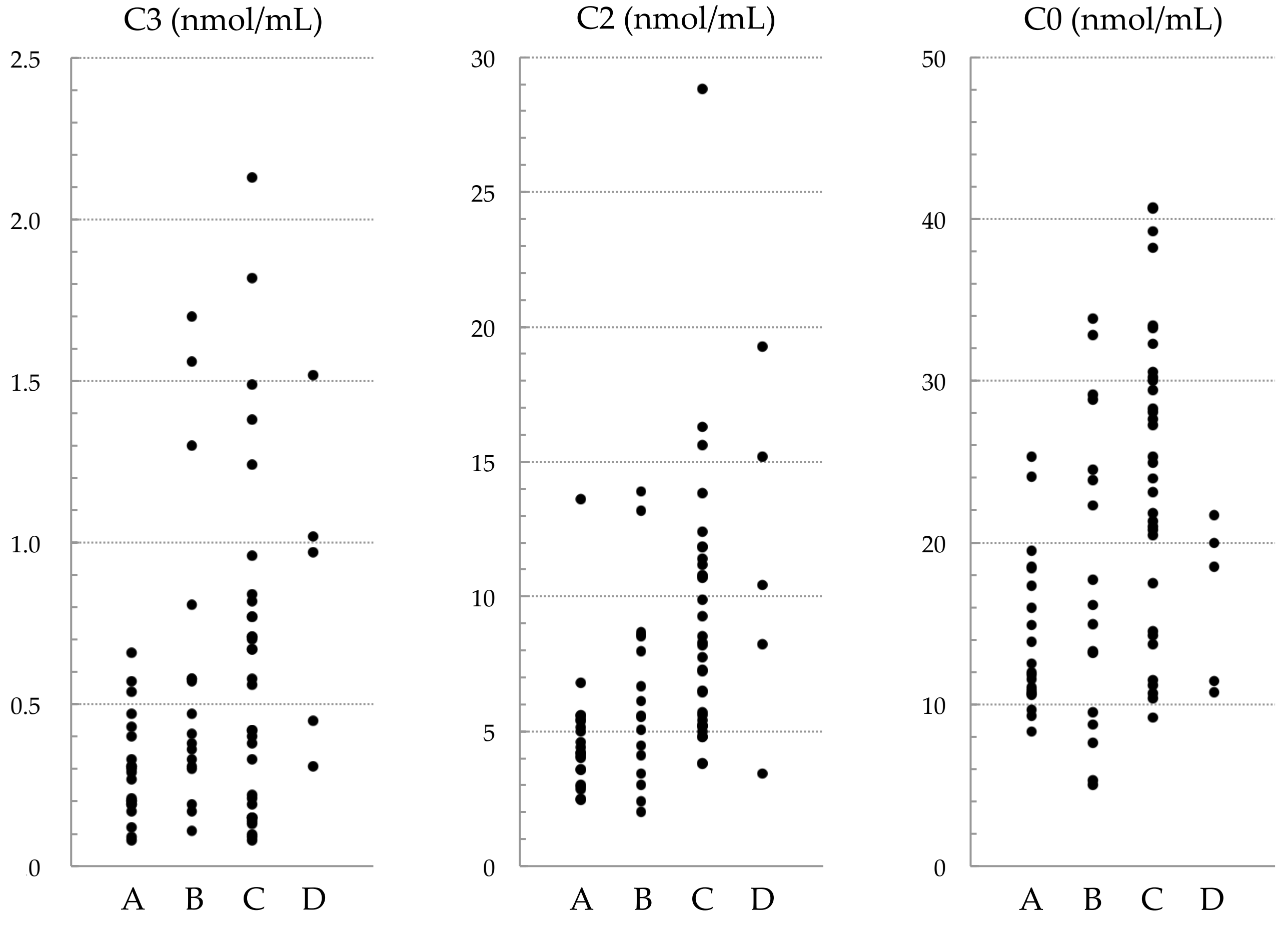

Figure S1. Concentrations of acylcarnitines and free carnitine (C0) in dried blood specimens from newborns. A–C: Newborn screening (NBS)–positive infants (A: patients with carnitine palmitoyltransferase (CPT) II deficiency; B: carriers heterozygous for pathogenic variants, infants homozygous for p.F352C, and other infants with carrier-level CPT II activity; and C: infants with normal-level CPT II activity). D: Patients with symptoms but normal NBS results.
