# Supplementary material for: Newborn Screening with (C16 + C18:1)/C2 and C14/C3 for Carnitine Palmitoyltransferase II Deficiency throughout Japan Has Revealed C12/C0 as an Index of Higher Sensitivity and Specificity
Source: Int J Neonatal Screen. 2023 Oct 27;9(4):62. doi: 10.3390/ijns9040062 (PMC10660675; doi:10.3390/ijns9040062)
Supplement: Supplementary file 1 [file IJNS-09-00062-s001.zip › IJNS2023_TableS1.pdf]

Table S1. Results of newborn screening (NBS) and confirmatory tests of the infants enrolled in this study.

Case ID	NBS Indices and Cutoff (Mean ± SD of 99.9th Percentile Values in 35 Laboratories)		Serum Acylcarnitine Levels (nmol/mL)		CPT II Activity (%)[1]	*CPT2* Genotype (Rare Variants and Thermolabile Polymorphisms)				Diagnoses of NBS-Positive Infants
	(C16 + C18:1)/C2 0.432 ± 0.073	C14/C3 0.386 ± 0.077	C16	C18:1		Variant 1	Variant 2	c.1055T > G p.F352C	c.1102G > A p.V368I	
NBS-positive infants										
S-01	0.75	0.86	3.01	3.92	13.6	c.1148T > A p.F383Y	c.481C > T p.R161W	F/C	V/I	Hypoglycemic encephalopathy[3]
N-01	2.108	3.16	1.60	1.48	1.2	c.1148T > A p.F383Y	c.1148T > A p.F383Y	C/C	I/I	Patient
N-02	3.26	4.26	3.02	3.21	3.3[2]	c.520G > A p.E174K	c.1148T > A p.F383Y	ND		Patient
N-03	1.40	3.18	2.17	2.41	3.3[2]	c.520G > A p.E174K	c.1148T > A p. F383Y	ND		Patient
N-04	1.14	2.20	0.84	0.61	4.1	c.520G > A p.E174K	c.656G > A p.R219Q	F/C	V/I	Patient
N-05	1.65	4.56	1.57	1.32	4.4[2]	c.1148T > A p.F383Y	c.1429C > T p.R477W	ND		Patient
N-06	1.10	1.72	0.65	0.88	4.4[2]	c.1511C > T p.P504L	c.1813G > C p.V605L	F/C	V/I	Patient
N-07	3.44	5.65	3.18	2.54	4.8[2]	c.451C > T p.R151W	c.1148T > A p. F383Y	F/C	I/I	Patient
N-08	0.47	0.69	0.32	0.28	6.4	c.214_215delTT p.L72Efs	c.1891C > T p.R631C	F/C	V/I	Patient
N-09	1.31	1.12	2.25	2.37	7.5	c.520G > A p.E174K	c.1148T > A p.F383Y	F/C	V/I	Patient
N-10	3.01	ND	3.06	4.24	7.8	c.1121G > A p.W374*	c.1148T > A p.F383Y	F/C	V/I	Patient
N-11	0.86	0.93	0.83	2.15	8.7	c.1115A > G p.H372R	c.1508G > A p.R503H	F/C	I/I	Patient
N-12	3.27	8.25	1.47	2.46	12.1	c.1148T > A p.F383Y	c.1148T > A p.F383Y	C/C	I/I	Patient
N-13	0.55	1.00	0.61	0.87	12.4	c.1492C > T p.R498C	c.1813G > C p.V605L	C/C	I/I	Patient
N-14	6.31	4.89	5.65	5.00	13.4	c.520G > A p.E174K	c.1737delC p.Y579*	F/F	V/V	Patient
N-15	4.60	8.39	ND	ND	14.2	c.520G > A p.E174K	c.1737delC p.Y579*	F/F	V/V	Patient
N-16	0.56	1.01	0.68	0.82	15.8	c.520G > A p.E174K	c.1813G > C p.V605L	F/C	V/I	Patient
N-17	0.87	1.75	2.29	3.00	16.5	c.1148T > A p.F383Y	c.1148T > A p.F383Y	C/C	I/I	Patient
N-18	0.50	0.98	0.41	0.33	19.9	c.520G > A p.E174K	c.1813G > C p.V605L	F/C	V/I	Patient
N-19	0.87	1.26	0.70	0.57	20.7	c.106C > T p.Q36*	c.1813G > C p.V605L	C/C	V/V	Patient
N-20	0.67	1.58	1.70	1.70	27.9	c.520G > A p.E174K	c.1813G > C p.V605L	F/C	V/I	Patient
N-21	0.51	0.40	0.23	0.25	31.8	c.1525A > G p.T509A	None	F/C	I/I	Heterozygous carrier
N-22	0.52	0.69	0.18	0.20	34.0	None	None	C/C	I/I	p.F352C homozygote
N-23	0.44	0.45	0.17	0.12	37.7	Not analyzed				Heterozygous carrier?
N-24	0.44	0.52	0.17	0.28	44.3	None	None	F/C	V/I	Normal?
N-25	1.54	0.26	0.07	0.08	51.3	None	None	F/F	V/I	Normal?
N-26	0.77	0.39	0.29	0.15	52.0[2]	None	None	C/C	I/I	p.F352C homozygote
N-27	0.48	0.12	0.13	0.17	52.0	None	None	F/F	I/I	Normal?
N-28	0.44	0.47	0.15	0.16	53.0	None	None	F/C	V/V	Normal?
N-29	0.45	0.39	0.09	0.10	53.6	Not analyzed				Heterozygous carrier?
N-30	0.68	0.25	0.10	0.09	54.8	Not analyzed				Heterozygous carrier?
N-31	0.52	1.35	ND	ND	58.8	Not analyzed				Heterozygous carrier?
N-32	0.59	0.23	0.09	0.10	59.1	Not analyzed				Heterozygous carrier?
N-33	0.34	0.40	0.21	0.27	59.9	Not analyzed				Heterozygous carrier?
N-34	1.21	0.54	0.06	0.21	63.6	Not analyzed				Heterozygous carrier?
N-35	0.45	0.35	0.19	0.19	63.8	None	None	C/C	V/V	p.F352C homozygote
N-36	0.64	0.18	0.17	0.24	67.4[2]	None	None	F/C	I/I	Normal?
N-37	0.84	0.17	0.17	0.06	70.8	c.1634A > C p.E545A	None	F/F	V/I	Heterozygous carrier
N-38	0.48	2.50	0.10	0.09	78.1	Not analyzed				Normal

N-39	0.45	0.44	0.10	0.35	92.6	None	None	F/F	V/V	Normal
N-40	0.45	0.41	0.08	0.22	93.0		Not analyzed			Normal
N-41	0.77	0.42	0.04	0.09	95.8		Not analyzed			Normal
N-42	0.55	1.40	0.13	0.17	98.0	None	None	F/F	V/V	Normal
N-43	0.84	0.37	0.09	0.07	99.3 [2]	None	None	F/C	I/I	Normal
N-44	0.70	0.46	0.17	0.16	100.0 [2]	None	None	F/F	V/V	Normal
N-45	0.45	0.41	0.06	0.12	101.4		Not analyzed			Normal
N-46	0.48	0.36	0.09	0.05	102.4		Not analyzed			Normal
N-47	0.69	0.19	ND	ND	108.1		Not analyzed			Normal
N-48	0.59	1.83	0.23	0.07	109.1	None	None	F/F	V/I	Normal
N-49	1.10	0.92	0.08	0.09	109.2	None	None	F/F	I/I	Normal
N-50	0.65	0.11	0.09	0.09	113.9 [2]	None	None	F/C	I/I	Normal
N-51	0.45	1.24	0.22	0.21	116.8	None	None	F/C	I/I	Normal
N-52	0.52	0.37	0.16	0.13	116.9		Not analyzed			Normal
N-53	0.45	0.39	0.10	0.08	120.1		Not analyzed			Normal
N-54	0.57	0.22	ND	ND	122.0		Not analyzed			Normal
N-55	0.83	0.25	0.09	0.12	126.0 [2]		Not analyzed			Normal
N-56	0.52	0.29	0.06	0.06	128.0		Not analyzed			Normal
N-57	0.43	0.37	0.19	0.14	132.5		Not analyzed			Normal
N-58	0.45	1.89	0.15	0.11	141.2		Not analyzed			Normal
N-59	0.76	0.44	0.08	0.05	144.0	None	None	F/F	I/I	Normal
N-60	0.58	1.22	0.08	0.09	144.3	None	None	F/F	V/I	Normal
N-61	0.57	0.17	0.09	0.05	152.7		Not analyzed			Normal
N-62	0.56	0.38	0.09	0.10	153.7		Not analyzed			Normal
N-63	0.46	0.30	0.11	0.10	153.9 [2]	None	None	F/C	I/I	Normal
N-64	0.40	0.38	0.06	0.03	156.6		Not analyzed			Normal
N-65	0.46	0.37	ND	ND	167.7		Not analyzed			Normal
N-66	0.64	0.19	0.09	0.11	195.6 [2]		Not analyzed			Normal
N-67	0.46	0.39	0.17	0.15	258.6	None	None	F/F	V/I	Normal
N-68	0.48	0.55	0.15	0.11	266.3		Not analyzed			Normal
N-69	0.56	1.27	0.05	0.05	389.2		Not analyzed			Normal
N-70	0.76	2.01	0.08	0.06	410.4		Not analyzed			Normal
Infants with symptoms but normal NBS results										Clinical symptom
S-02	0.32	0.22	0.64	0.71	8.1	c.338C > T p.S113L	c.1933G > T p.E645*	F/C	V/I	Rhabdomyolysis
S-03	0.31	0.36	0.69	0.65	15.5	c.1678C > T p.R560W	c.1813G > C p.V605L	C/C	I/I	Rhabdomyolysis
S-04	0.15	0.24	1.17	1.88	16.2	c.1148T > A p.F383Y	c.1678C > T p.R560W	C/C	I/I	Rhabdomyolysis
S-05	0.22	0.27	0.95	1.27	19.0		Not analyzed			Rhabdomyolysis
S-06	0.20	0.09	1.74	2.40	30.2	c.365C > T p.S122F	c.520G > A p.E174K	F/F	V/V	Rhabdomyolysis

Abbreviations: carnitine palmitoyltransferase II, CPT II; no data, ND; homozygous for p.F352C C/C; heterozygous for p.F352C, F/C; homozygous for p.F352, F/F; homozygous for p.V368I, I/I; heterozygous for p.V368I, V/I; homozygous for p.V368, V/V.

Partial data for S-01, N-02, N-03, N-05, N-06, N-07, N-10, N-12, N-21, N-26, N-36, N-37, N-43, N-47, N-50, N-52, N-54, N-55, N-56, N-61, N-63, and N-66 were presented in a previous report [4].

Partial data for S-01, N-02, N-03, N-04, N-05, N-06, N-10, N-12, N-20, N-21, N-37, S-02, S-03, and S-04 were presented in a previous report [6].

[1] Mean reference value of CPT II activity in 85 normal controls is 126.7 ± 43.2 pmol/min/10^5 lymphocytes.

[2] CPT II activity is calculated from d_{27}C14/d_{31}C16 in fatty acid oxidation assay: mean reference value in 36 normal controls is 0.273 ± 0.096.

[3] S-01 was a patient with symptoms who had passed our pilot NBS with C16 and C18:1; his data one reason to adopt (C16 + C18:1)/C2.
