# Supplementary material for: Newborn Screening with (C16 + C18:1)/C2 and C14/C3 for Carnitine Palmitoyltransferase II Deficiency throughout Japan Has Revealed C12/C0 as an Index of Higher Sensitivity and Specificity
Source: Int J Neonatal Screen. 2023 Oct 27;9(4):62. doi: 10.3390/ijns9040062 (PMC10660675; doi:10.3390/ijns9040062)
Supplement: Supplementary file 1 [file IJNS-09-00062-s001.zip › IJNS2023_TableS2.pdf]

Table S2. Concentrations of acylcarnitines and free carnitine (C0) in dried blood specimens from newborns.

Case ID	Acylcarnitine Levels in Dried Blood Specimen from Newborns (nmol/mL)													
	C16	C18:1	C18	C16-OH	C14	C14:1	C12	C10	C8	C6	C4	C3	C2	C0
S-01	3.45	1.68	1.47	0.041	0.57	0.14	0.46	0.29	0.09	0.04	0.16	0.66	6.81	25.31
N-01	3.46	2.57	1.71	0.04	0.60	0.11	0.98	0.25	0.08	ND	ND	0.19	2.86	13.88
N-02	12.20	6.05	4.13	0.08	1.32	0.14	0.55	0.18	0.07	0.03	0.12	0.31	5.60	14.90
N-03	13.07	5.92	4.41	0.25	1.05	0.38	0.46	0.34	0.16	0.10	0.10	0.33	13.60	19.50
N-04	1.85	0.96	0.73	UD	0.26	0.07	0.17	0.08	0.03	0.01	0.03	0.12	2.46	11.08
N-05	5.07	1.54	1.24	0.08	0.41	0.13	0.16	0.23	0.08	0.04	0.06	0.09	4.01	10.89
N-06	3.37	2.31	1.10	0.031	0.74	0.16	0.60	0.22	0.07	0.01	0.07	0.43	5.16	8.32
N-07	9.93	5.25	3.27	0.06	1.75	ND	1.04	0.29	0.06	0.02	ND	0.31	4.40	18.50
N-08	1.07	0.90	0.57	0.009	0.21	0.05	0.32	0.09	0.07	0.02	0.09	0.30	4.20	11.80
N-09	4.47	2.81	2.18	0.032	0.64	0.069	0.389	0.107	0.035	ND	ND	0.57	5.56	24.05
N-10	10.60	4.45	1.40	0.10	ND	0.20	1.00	0.30	0.10	0.02	ND	0.20	5.00	12.00
N-11	2.76	1.25	0.75	0.029	0.36	0.09	0.20	0.12	0.10	0.02	0.09	0.40	4.60	12.50
N-12	4.98	3.22	1.96	0.04	0.66	0.12	0.32	0.12	0.03	0.02	0.04	0.08	2.51	11.55
N-13	1.80	1.20	0.55	0.03	0.54	0.05	0.50	0.17	0.04	0.02	0.07	0.54	5.44	18.41
N-14	17.20	5.41	4.28	0.186	1.40	0.17	0.34	0.09	0.03	0.02	0.05	0.29	3.58	10.60
N-15	17.80	6.91	4.76	0.225	1.75	0.13	0.855	0.125	0.04	0.002	0.051	0.21	5.37	15.97
N-16	1.29	1.00	0.54	0.02	0.50	0.06	0.49	0.11	0.04	0.02	0.11	0.47	4.09	17.34
N-17	1.38	1.23	0.60	0.02	0.30	0.06	0.73	0.36	0.11	ND	ND	0.17	3.00	9.29
N-18	0.89	0.59	0.39	0.013	0.20	0.05	0.20	0.07	0.02	0.01	0.05	0.20	2.96	10.75
N-19	2.62	1.03	0.65	0.028	0.33	0.066	0.228	0.063	0.031	0.013	0.046	0.27	4.19	9.69
N-20	1.26	1.17	0.60	0.015	0.28	0.04	0.52	0.14	0.05	0.01	0.10	0.19	3.61	10.65
N-21*	2.04	0.72	0.50	0.006	0.10	0.05	0.09	0.08	0.08	ND	ND	0.47	6.67	8.74
N-22	1.15	0.63	0.37	0.01	0.08	0.02	0.02	0.01	0.01	ND	ND	0.11	3.42	9.50
N-23	2.53	1.21	0.62	0.01	0.19	0.04	0.07	0.07	0.09	0.02	0.12	0.41	8.60	22.30
N-24	0.85	0.47	0.27	UD	0.17	0.01	0.05	0.03	0.02	UD	0.04	0.33	3.00	29.14
N-25	2.32	1.44	0.96	0.01	0.15	0.027	0.035	0.023	0.026	0.007	0.09	0.57	2.40	32.80
N-26	3.51	1.18	1.33	0.025	0.15	0.063	0.076	0.099	0.073	0.046	0.123	0.38	6.13	13.27
N-27	4.48	2.22	1.44	0.02	0.19	0.06	0.10	0.07	0.06	0.03	ND	1.70	13.89	14.98
N-28	1.36	0.44	0.49	UD	0.09	0.02	0.03	0.02	0.01	0.01	0.05	0.19	4.11	7.63
N-29	1.24	1.23	0.56	0.01	0.14	ND	0.02	0.01	0.01	0.01	0.10	0.36	5.53	13.20
N-30	2.61	0.82	0.46	0.011	0.14	0.04	0.02	0.04	0.01	0.01	0.09	0.58	5.06	17.69
N-31	1.78	1.11	0.38	0.01	0.23	0.05	0.08	0.07	0.08	0.02	0.05	0.17	5.56	33.82
N-32	3.39	1.35	0.74	0.017	0.18	0.05	0.07	0.119	0.063	0.034	0.16	0.81	7.98	23.84
N-33	0.86	0.64	0.43	0.01	0.12	0.04	0.03	0.03	0.04	ND	ND	0.31	4.48	5.30
N-34	1.40	0.80	0.60	UD	0.10	ND	ND	ND	ND	ND	ND	0.30	2.00	5.00
N-35	4.40	1.49	0.94	0.03	0.20	0.11	0.12	0.11	0.07	ND	ND	0.58	13.18	28.80
N-36	3.41	2.00	1.47	0.02	0.23	0.061	0.101	0.066	0.045	0.031	0.16	1.30	8.52	16.15
N-37	6.11	1.20	1.29	0.04	0.26	0.15	0.14	0.11	0.07	0.01	0.15	1.56	8.67	24.51
N-38	1.64	1.49	0.36	0.01	0.20	0.07	0.08	0.07	0.08	0.01	0.04	0.08	6.51	40.63
N-39	2.53	1.20	0.80	0.02	0.31	0.05	0.07	0.07	0.03	0.01	0.10	0.71	8.28	39.22
N-40	4.05	1.47	0.76	0.05	0.34	0.22	0.24	0.28	0.17	0.01	0.07	0.84	12.41	30.54
N-41	1.86	1.05	0.74	0.01	0.14	0.02	0.02	0.02	0.05	0.01	0.06	0.33	3.79	17.49
N-42	2.69	1.33	0.64	0.02	0.26	0.10	0.08	0.11	0.08	0.02	0.04	0.19	7.30	38.20
N-43	5.18	1.93	1.54	0.024	0.24	0.101	0.094	0.081	0.044	0.009	0.149	0.77	8.51	30.25

N-44	3.33	2.12	1.25	0.01	0.10	0.05	0.06	0.12	0.09	0.03	0.07	0.22	7.73	13.72
N-45	3.86	1.40	1.01	0.02	0.23	0.09	0.10	0.11	0.09	ND	ND	0.56	11.81	11.20
N-46	3.33	1.44	0.90	0.02	0.29	0.10	0.12	0.18	0.12	0.05	0.18	0.82	9.88	23.99
N-47	2.64	1.27	0.70	0.013	0.13	0.045	0.054	0.077	0.06	0.017	0.077	0.67	5.70	20.45
N-48	2.89	1.36	0.61	0.02	0.27	0.10	0.12	0.16	0.09	ND	ND	0.15	7.21	29.40
N-49	2.80	1.64	1.04	0.009	0.14	0.04	0.07	0.06	0.04	0.02	0.05	0.15	3.81	9.16
N-50	4.44	1.55	1.18	0.017	0.20	0.062	0.064	0.055	0.033	0.009	0.246	1.82	9.26	27.27
N-51	1.19	1.12	0.45	0.01	0.19	0.05	0.06	0.05	0.04	0.01	0.03	0.15	5.20	25.30
N-52	4.04	1.75	1.28	0.02	0.26	0.14	0.11	0.18	0.11	0.04	0.12	0.70	11.18	24.92
N-53	2.74	0.96	0.59	0.015	0.11	0.06	0.04	0.05	0.03	0.02	0.14	0.38	8.18	11.51
N-54	3.74	2.35	1.23	0.021	0.21	0.069	0.105	0.119	0.062	0.034	0.19	0.96	10.79	14.25
N-55	3.11	1.39	0.95	0.01	0.17	ND	ND	ND	ND	ND	ND	0.67	5.42	21.31
N-56	6.06	2.10	1.37	0.038	0.40	0.22	0.15	0.18	0.09	0.03	0.20	1.38	15.60	23.10
N-57	2.58	1.97	0.79	0.01	0.26	0.01	0.04	0.03	0.03	0.01	0.15	0.71	10.68	40.68
N-58	1.48	0.77	0.35	0.01	0.17	0.07	0.06	0.40	0.09	0.01	0.04	0.09	4.99	20.78
N-59	3.08	1.19	0.66	0.014	0.19	0.066	0.071	0.058	0.044	ND	ND	0.42	5.62	32.26
N-60	2.12	0.90	0.45	0.014	0.16	0.068	0.044	0.031	0.032	0.018	0.042	0.13	5.23	27.63
N-61	6.79	2.55	1.68	0.02	0.36	0.23	0.19	0.17	0.12	0.04	0.17	2.13	16.30	28.27
N-62	2.82	0.77	0.53	0.015	0.16	0.044	0.04	0.06	0.039	0.024	0.093	0.42	6.44	10.70
N-63	9.67	3.72	2.30	0.033	0.45	0.139	0.209	0.153	0.081	0.073	0.278	1.49	28.82	33.26
N-64	1.32	0.57	0.46	0.002	0.08	0.04	0.05	0.04	0.04	0.02	0.07	0.21	4.78	10.34
N-65	3.78	1.73	1.16	0.015	0.28	0.07	0.07	0.06	0.05	0.04	0.36	0.77	11.84	28.06
N-66	5.33	1.59	1.13	0.024	0.20	0.085	0.068	0.06	0.05	0.019	0.112	1.24	10.76	33.41
N-67	4.21	2.17	0.89	0.03	0.23	0.08	0.07	0.07	0.06	0.05	0.20	0.58	13.84	14.53
N-68	3.72	1.74	1.10	0.016	0.22	0.08	0.08	0.08	0.05	0.02	0.12	0.40	11.40	21.83
N-69	1.59	1.09	0.41	0.01	0.17	0.08	0.12	0.12	0.08	0.02	0.08	0.14	4.78	21.00
N-70	2.60	1.30	0.55	0.02	0.19	0.06	0.09	0.06	0.05	0.02	0.05	0.10	5.17	30.04
S-02	3.94	0.96	0.85	0.024	0.23	0.07	0.08	0.10	0.06	0.03	0.16	1.02	15.20	18.50
S-03	0.67	0.37	0.37	0.01	0.11	0.05	0.14	0.06	0.04	0.01	0.06	0.31	3.42	11.45
S-04	0.65	0.57	0.29	0.01	0.11	0.03	0.17	0.10	0.06	0.03	0.12	0.45	8.23	20.00
S-05	2.65	1.51	0.95	0.01	0.26	0.08	0.19	0.22	0.10	0.06	0.19	0.97	19.27	21.72
S-06	1.35	0.78	0.36	0.008	0.13	0.07	0.11	0.10	0.07	0.02	ND	1.52	10.42	10.76

Abbreviations: no data, ND; undetectable, UD.

Partial data for S-01, N-02, N-03, N-05, N-06, N-07, N-10, N-12, N-21, N-26, N-36, N-37, N-43, N-47, N-50, N-52, N-54, N-55, N-56, N-61, N-63, and N-66 were presented in a previous report [4].

Partial data for S-01, N-02, N-03, N-04, N-05, N-06, N-10, N-12, N-20, N-21, N-37, S-02, S-03, and S-04 were presented in a previous report [6].

*Newborn screening for N-21 was conducted in a regional laboratory, and then the same dried blood sample was sent to the University of Fukui for reanalysis. The data of this patient, presented in previous reports [4, 6], were obtained in the reanalysis, but the data for C14:1, C12, C10, C8, C6, and C4 tests are not available at present. Therefore, the data for N-21 in this study are substituted with those obtained in the first analysis.
